# Acoustic Environmental Conditions (Do Not?) Affect the Static Posturography Diagnostic Accuracy: A Test–Retest Reliability Study

**DOI:** 10.3390/s22062365

**Published:** 2022-03-18

**Authors:** Sofía Olivia Calvo-Moreno, Elena Sonsoles Rodríguez-López, Umut Varol, María Benito-de-Pedro, Elena Anós-Merino, Orlando Conde-Vázquez, César Fernández-de-las-Peñas, Juan Antonio Valera-Calero

**Affiliations:** 1 Department of Physiotherapy, Faculty of Health, Universidad Camilo José Cela, Villanueva de la Cañada, 28692 Madrid, Spain; socalvo@ucjc.edu (S.O.C.-M.); esrodriguez@ucjc.edu (E.S.R.-L.); mbenito@ucjc.edu (M.B.-d.-P.); elena.anos@alumno.ucjc.edu (E.A.-M.); 2 VALTRADOFI Research Group, Department of Physiotherapy, Faculty of Health, Universidad Camilo José Cela, Villanueva de la Cañada, 28692 Madrid, Spain; umut.varol@alumni.ie.edu; 3 Departamento de Biología Funcional y Ciencias de la Salud, Facultad de Fisioterapia, Universidad de Vigo, 36310 Vigo, Spain; orlando.conde@uvigo.es; 4 Department of Physical Therapy, Occupational Therapy, Rehabilitation and Physical Medicine, Universidad Rey Juan Carlos, Alcorcón, 28933 Madrid, Spain; cesar.fernandez@urjc.es; 5 Cátedra Institucional en Docencia, Clínica e Investigación en Fisioterapia: Terapia Manual, Punción Seca y Ejercicio Terapéutico, Universidad Rey Juan Carlos, Alcorcón, 28933 Madrid, Spain

**Keywords:** diagnostic accuracy, reliability, postural balance, stabilometry, noise, environment

## Abstract

Static posturography assessed with force platforms is a procedure used to obtain objective estimates related to postural adjustments. However, controlling multiple intrinsic and extrinsic factors influencing the diagnostic accuracy is essential to obtain reliable measurements and recommend its use with clinical or research purposes. We aimed to analyze how different environmental acoustic conditions affect the test–retest reliability and to analyze the most appropriate number of trials to calculate a valid mean average score. A diagnostic accuracy study was conducted enrolling 27 healthy volunteers. All procedures were taken considering consistent device settings, posture, feet position, recording time, and illumination of the room. Three trials were recorded in a silent environment (35–40 dB) and three trials were recorded in a noisy environment (85–90 dB). Results showed comparable reliability estimates for both acoustic conditions (ICC = 0.453–0.962 and 0.621–0.952), but silent conditions demonstrated better sensitivity to changes (MDC = 13.6–76%). Mean average calculations from 2 and 3 trials showed no statistically significant differences (*p* > 0.05). Cross-sectional studies can be conducted under noisy or silent conditions as no significantly different scores were obtained (*p* > 0.05) and ICC were comparable (except oscillation area). However, longitudinal studies should consider silent conditions as they demonstrated better sensitivity to real changes not derived from measurement errors.

## 1. Introduction

Balance could be defined as the result of postural adjustments regulated by a complex system of mechanisms involving several multisensory inputs (e.g., visual, vestibular, auditive, and somatosensory) [1] and aims to keep the static postural control, defined as the ability to achieve and maintain the center of mass within the base of support correcting disturbances [2]. Although these inputs depend directly on the afference processing at the central nervous system, several intrinsic and extrinsic factors are continuously altering this balance [3]. Hence, continuous voluntary and automatic efferent responses adjust the motor output to stabilize the body in a 4-step process: stimulation of sensory receptors, afferent signaling via sensory neurons, information processing and decision making at the central nervous system, and efferent signaling to skeletal muscles via alpha-motoneurons [4].

Since postural control depends on a combination of passive (i.e., stiffness and kinematic properties associated with bones, ligaments and joints) and active (i.e., interactions between skeletal muscles and both the peripheral and central nervous system) mechanical controls, previous studies analyzed how sensory (i.e., visual, vestibular, auditive, and somatosensory) afferences influence balance disturbances in athletes [5], elderly [6] and clinical populations [7,8].

Sensor applications in healthcare have been widely developed in recent years [9]. Static posturography (assessed with stabilometry) is a widely used, fast and non-invasive tool that provides sensitive information about different parameters related with the oscillation of the center of gravity (expressed with speed or distance units) obtained during a static standing position on a force platform [10]. Since this tool allows the examiner to obtain multiple objective parameters in contrast with other screening tests (e.g., Y-Balance, Bucket Fukuda Stepping or Romberg tests) [11], it is considered a Gold Standard [12]. However, its utility (i.e., validity, reliability, sensitivity, and specificity) depends on strict and consistent protocols to ensure its reproducibility.

Previous studies considered standardization of intrinsic factors including the standing posture during the record [13,14] focusing on the position of the feet [14,15] and the calibration of the instrument including the sampling frequency [13,15] and recording time [3,14,16]. Although environmental conditions are crucial as well as obtaining reliable measurements, there is limited evidence analyzing how the environment could induce measurement bias and most of the authors focused on sight aspects (e.g., size, distance and placement of the visual target and illumination) [14].

Since it has been demonstrated that hearing capacity is a determinant factor for improving postural stability [17] our aims were: (1) to analyze the test–retest reliability of static postugraphy under two different acoustic conditions (noisy and silent environment) and (2) to analyze the most efficient number of trials (considering up to three measurements) needed to provide reliable results in the balance scores.

## 2. Materials and Methods

### 2.1. Study Design

A diagnostic accuracy study was conducted to assess validity and reliability estimates for stabilometric assessment considering the influence of the number of trials and how different acoustic environmental conditions influence the measurement of oscillation surface area, oscillation length along both lateral and anteroposterior axes, total oscillation length, comparison of length of movement and covered area, relation between the gravity center speed movement and the average movement on the anteroposterior axis, average of speed variation, and mean speed.

This study followed the recommendations from the Guidelines for Reporting Reliability and Agreement Studies (GRRAS) [18] and the Enhancing the QUAlity Transparency Of health Research (EQUATOR) guidelines [19]. The study protocol was supervised and approved by the Institutional Ethics Committee of Clinical Research of Camilo José Cela University (UCJC 05-12-2018) and conducted in accordance with the Declaration of Helsinki.

### 2.2. Participants

A consecutive sample of healthy participants was screened for eligibility criteria by using local announcements posted around the University from September 2021 to December 2021. To be eligible for participation, volunteers had to be aged between 18 and 65 years and read and sign the written informed consent prior to their inclusion. Exclusion criteria included presence of neurological diseases, somatosensory disorders, eye diseases (e.g., corneal diseases, pupil disorders, retinal diseases, or refractive errors) or any other condition affecting the balance, afferences processing or the vestibular system (e.g., vertigo, vestibulopathy or semicircular canal dehiscence). Participants were required not to perform intense physical activity the day before and had to rest for between 7 and 8 h the previous night as recommended [16].

### 2.3. Sample Size Calculation

Sample size calculation was estimated following the directions described by Walter et al. [20]. Based on the intraclass correlation coefficient (ICC) obtained in previous reliability studies [3,16,21,22,23], an ICC > 0.7 (ρ_0_) was set as the minimal acceptable value and ICC > 0.9 (ρ_1_) was set as the expected value. For the sample size calculation, a power of 80% and a significance level of 5% were set.

Therefore, since test–retest reliability was calculated as the mean average from two and three trials per patient, assuming an estimated 10% loss (due to the longitudinal nature of this test–retest study design), a minimum sample size of 26 (for mean average obtained from two trials) and 19 (for mean average obtained from three trials) participants, respectively, could be considered appropriate.

### 2.4. Procedures

The static posturography assessments were conducted 3 times considering 2 different environmental conditions (noisy and silent conditions) to analyze the influence of environmental noise on the diagnostic accuracy. The noise intensity in the room was controlled using the NIOSH Sound Level Meter (EA LAB, Slovenia) app for iOS (Apple, Cupertino, CA, USA) as demonstrated to be an acceptable and reliable method for measuring noise exposure levels (R^2^ = 0.97) [24]. Two speakers located laterally and behind the participant’s position played an 8D street ambience noise during the procedure in order to avoid acoustic spatial orientation. 8D sounds are based on the Haas effect, and it consists of adjusting sound frequencies to simulate sounds “coming” from different directions to avoid acoustic directional orientation. Silent environment was set at a range of 35–40 dB and noisy environment was set at a range of 85–90 dB (measured at the participant’s ears position). The starting acoustic condition was randomly selected using a random number generator (Research Randomizer Vr.4.0) in order to reduce the between-trials habituation influence.

According to the current standards reported by Yamamoto et al. [14], we considered several intrinsic and extrinsic factors. Device settings (Fusyo stabilometric platform—Medicapteurs trademark, France—setting a 40 Hz sampling frequency as is demonstrated to be adequate for obtaining valid and reliable measurements [16]), posture (participants were placed in a relaxed and natural standing posture with arms extended laterally), position of the feet (each foot placed at 30°, no contact between the contralateral heels and without socks and footwear), eyes closed, recording time (30 s as recommended), target distance and size (in order to standardize participants’ positions before starting the measurements with closed eyes, a circular 3 cm target was placed on a wall situated 1 m in front of the subject at eye level) and illumination of the room (around 50 lx) were consistent for all the measurements.

Although jaw position was shown to not be relevant in healthy people or patients with temporomandibular disorders [25], we decided to make the procedure consistent by asking the patients to not clench their jaw. The procedure was canceled and repeated if the patient coughed, sneezed, yawned, turned their head, or any other movement involving a loss of the position indicated previously.

**Figure 1:**
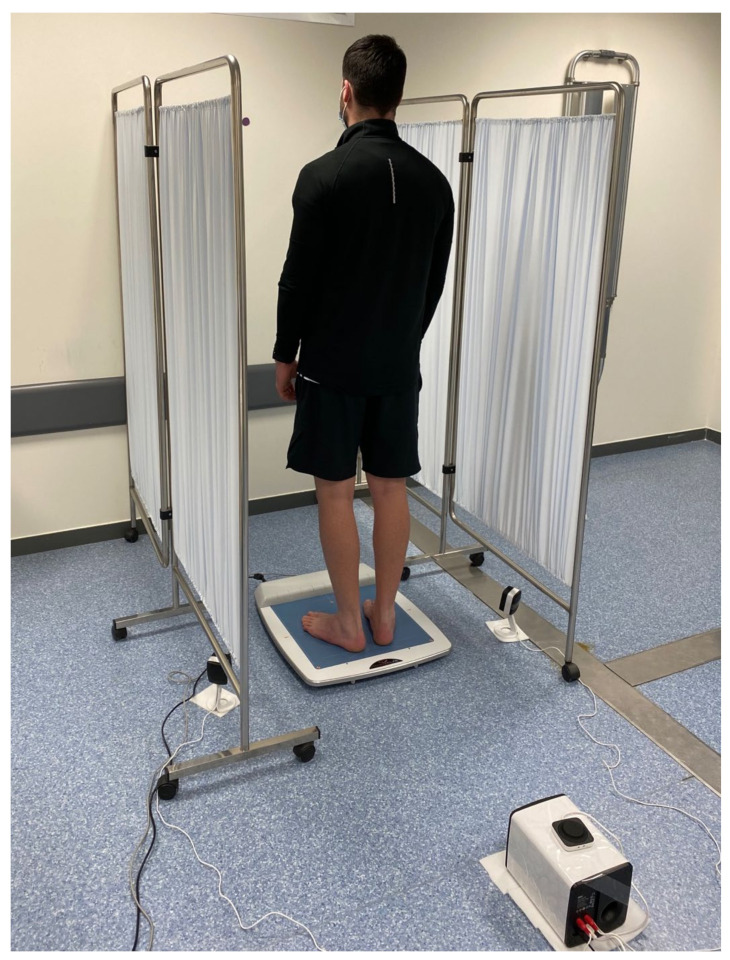
Participants’ stabilometric assessment and environmental conditions.

All the procedures were carried out between 9 a.m. and 1 p.m. and conducted in the nursery simulator room located in Camilo José Cela University (Madrid, Spain) by two experienced raters who were familiar with the instrument and the software (Figure 1).

### 2.5. Variables

Sociodemographic data was collected using a self-reported clinical history asking for the participants’ age, gender, height, weight, body mass index (BMI), and foot size.

**Figure 2:**
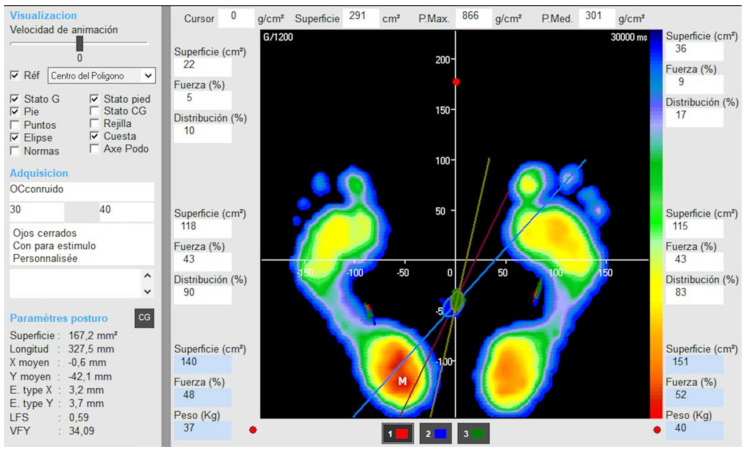
Software used for measuring dynamic balance outcomes.

Stabilometric parameters registered were the oscillation surface area, absolute oscillation length in both the medio-lateral and antero-posterior directions, total oscillation length, comparison of length of movement and covered area (length as a function of the surface or LFS), quotient between the velocity at which the center of gravity is moving and the movement along the anteroposterior axis (speed variation as a function of Y or SFVAP), average of speed variation (ASV), and mean speed. All scores were automatically generated in the software after each trial (Figure 2).

### 2.6. Statistical Analysis

All statistical analyses were performed using the Statistical Package for the Social Science (SPSS) software v.27 (IBM, Armonk, NY, USA) for Mac OS. All tests were two-tailed and a 5% significance level (*p* < 0.05) was set. Firstly, the baseline sample data distribution for continuous variables was verified using the Shapiro–Wilk test (*p* > 0.05).

Test–retest reliability was assessed for each stabilometric parameter under the two environmental acoustic conditions proposed considering the mean average of 3 trials using consistency-type, two-way mixed-model intraclass correlation coefficients (ICC_2,1_) [26]. ICC cut-off scores were interpreted as reported by Koo and Li [26]: poor reliability (ICC < 0.39); fair reliability (0.40 < ICC < 0.69); good reliability (0.70 < ICC < 0.89); and excellent (0.90 < ICC). In addition, absolute error between trials 1 and 2, standard error of measurement (SEM = standard deviation of difference score * √1−ICC), minimal detectable changes (MDC_95_ = SEM × √2 × 1.96), and coefficient of variation (CV% = Standard Deviation/Mean) were calculated. Finally, Student’s T-tests were used to analyze score differences between conducting the mean average of 2 and 3 trials and differences between both acoustic conditions (based on the average of 3 trials).

## 3. Results

From a total of 34 volunteers initially enrolled in the study, 27 healthy participants were finally analyzed. Although all subjects met the inclusion and exclusion criteria, measurements from seven participants were tainted due to cancelled measurements in at least one trial (n = 4 sneezed, n = 2 coughed and n = 1 turned their head). Baseline characteristics of the sample are summarized in Table 1. Males exhibited greater foot size (*p* < 0.001), height (*p* < 0.001), and weight (*p* = 0.030). However, BMI and age were comparable (*p* > 0.05).

**Figure 3:**
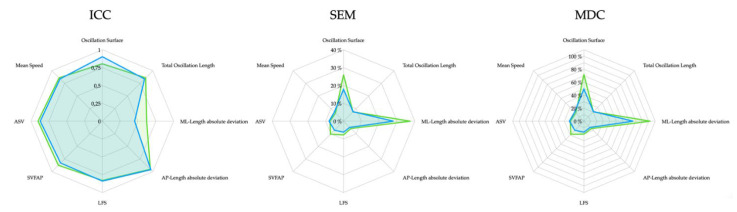
ICC_2,1_, SEM% and MDC_95_% scores for 35–40 dB (blue) and 85–90 dB (green).

Table 2 describes test–retest reliability estimates for each stabilometric parameter for 35–40 dB and 85–90 dB conditions. In general, most of the parameters showed good-to-excellent reliability for the silent (ICC ranged from 0.829 to 0.962) and the noisy (ICC ranged from 0.831 to 0.952) conditions. However, the mediolateral deviations showed to be fairly reliable. Although both acoustic conditions showed similar reliability estimates, the capacity of static posturography to detect whether changes are due to a real change and not due to a measurement error (based on MDC scores obtained) demonstrated noisy conditions to be in general somewhat less sensitive to changes than silent conditions. For instance, a minimum change of 72.2% in the oscillation surface area is needed to be attributable to a real change if measured in a noisy environment while a minimum change of 49.4% is needed if measured under silent conditions. MDC percentages in a silent environment ranged from 13.6% (for anteroposterior deviation length) to 76% (for the mediolateral deviation length). In noisy acoustic conditions, MDC percentages ranged from 16.1% (for anteroposterior deviation length) to 102.5% (for the mediolateral deviation length). Figure 3 compares test–retest reliability scores (ICC_2,1_, SEM% and MDC_95_%) between 35–40 dB and 85–90 dB conditions.

Table 3 describes how calculating the mean average of 2 or 3 measurements results in comparable scores for both acoustic conditions (*p* > 0.05).

Finally, Table 4 summarizes posturography score differences between silent and noisy environmental conditions. Results showed no statistically significant differences for all parameters assessed in this study (*p* > 0.05).

## 4. Discussion

The objectives of this study were to provide test–retest reliability estimates of static posturography parameters obtained in two acoustic environmental situations (silent and noisy conditions) and to calculate whether the mean average score obtained from two or three measurements differs for providing clinicians with accurate and efficient guidelines for measuring balance-related outcomes.

Our results suggested that acoustic conditions do not generally influence the test–retest reliability as ICC were comparable in both conditions. Although most of the parameters showed good-to-excellent ICC scores, the high intra-subject medio-lateral displacement variability resulted in fair reliability for this parameter in both acoustic conditions. Even if the ability to detect real changes not related to measurement errors was generally better in silent conditions, static posturography scores were comparable between both acoustic conditions. Therefore, we would recommend to control noise levels especially in longitudinal design studies. Regarding the mean average of two and three trial differences, we found no statistically significant scores. Therefore, calculating the mean average of two trials seems to be the most efficient option to reduce the burden with respect to time and cost.

Previous research used static posturography as a main outcome for multiple populations including athletes [27], elderlies [28] and patients with neurological conditions [7,8] seeking stabilometric factors associated with risk of falls, injuries or participants’ strength [29,30] or analyzing the effects of specific interventions on postural balance [31]. However, it is known that multiple intrinsic and extrinsic factors could affect balance disturbances and corrective responses [32,33,34,35]. In fact, in response to clinicians’ needs for accurate and valid methods assessing balance impairments, some articles aimed to identify and isolate each contributor to provide standardized guidelines [36,37,38,39].

Research and clinical consequences associated with reliable, valid, specific, and sensitive tools are evident since data collections are as valid as the instrument used. Therefore, both clinical and research findings may be misleading if measurement errors are too large or the ability to detect true changes is not sufficient [39].

Although the force platform is a single tool allowing the examiner to obtain a wide range of stabilometric parameters, not all of them showed to be equally reliable. For instance, medio-lateral imbalance amplitude showed a high intra-subject variability compared with the antero-posterior length, similarly to those results reported by Rodríguez-Rubio et al. [16]. A possible reason explaining the high intra-subject variability in the X axis is the small magnitude of this parameter. Therefore, small disturbances in this direction may result in exacerbated reliability loss. Further clinical research should consider the high intra-subject variability for this parameter for avoiding erroneous conclusions.

Another interesting finding is that a noisy environment affects both the test–retest reliability and the ability to detect real changes while assessing the sway imbalance area (considered as one of the most important parameters) [39]. Although final scores did not differ significantly between both conditions, controlling the acoustic conditions seems to be important to improve the diagnostic accuracy of static posturography (especially in those with longitudinal designs) as suspected [14].

Finally, average scores obtained from two and three trials were compared for assessing if the number of trials is a determinant factor in obtaining accurate data. Pineda et al. [3] reported that both ICC and SEM are closely associated with trial duration and number of repetitions (where shorter durations require a greater number of repetitions to maintain the reliability level). For instance, the authors reported that 15 repetitions of 30 s were needed for measuring the ellipse area and 4 repetitions of 30 s for measuring the medio-lateral and antero-posterior amplitude lengths in order to meet acceptable reliability. In contrast, our results showed comparable scores and reliability following the above procedure for two and three measurements, possibly due to a more specific control of internal and external factors which are potential factors increasing the within-subject variability of the body balance (from a neurophysiological point of view as explained in the introduction). Since more than three trials is not readily feasible during the clinical practice as this would significantly increase the patients’ and therapists’ burden, and since no differences between the average calculated from two and three trials were found, our recommendation is to perform the mean average of two trials as long as internal and external factors are controlled.

### Limitations

Some limitations should be acknowledged. First, the sample enrolled in this study was highly specific and healthy. We do not know if reliability estimates (ICC, SEM and MDC) would differ in clinical populations as different factors may affect in different manners for each specific condition and further research could analyze the clinical utility in clinical populations. Secondly, although we provided the minimal change to be attributable to a real change and not to a measurement error, we did not assess the magnitude of the change to be clinically relevant. Further longitudinal studies should estimate the minimal stabilometric change needed to be associated with a real clinical improvement. In addition, the number of trials was limited to three. We do not know if better reliability estimates would be obtained with a mean of +4 trials or if the instrument accuracy would be significantly increased. Finally, maybe a more feasible alternative to reduce the noise perceived by the participants is the use of earmuffs. Future studies could verify if reliability estimates are comparable and which strategy (use of earmuffs or controlling the entire room noise level) is more feasible during clinical practice.

## 5. Conclusions

Our results suggested that acoustic conditions do not generally influence static posturography scores or the test–retest reliability as ICC were comparable in both conditions. Although most of the parameters showed good-to-excellent ICC scores, the medio-lateral displacements demonstrated fair reliability either in silent or noisy environments. However, ICCs for oscillation area were slightly affected by the noise intensity, demonstrating excellent reliability in silent conditions and good in noisy conditions. Longitudinal studies should consider noise levels since the capacity of the force platforms for detecting real changes was generally better in silent conditions (lower MDC scores). Finally, the mean average obtained from 2 and 3 trials did not differ significantly. Therefore, reducing the number of trials from 3 to 2 (as long as internal and external factors are controlled) seems to be the most efficient choice in order to reduce time and costs.

## Figures and Tables

**Table 1:** Demographic and anthropometric characteristics of the sample.

Variables	Total Sample (n = 27)	Males (n = 12)	Females (n = 15)	Between Group Differences
Age (years)	20.1 ± 2.8	21.1 ± 3.8	19.3 ± 1.5	1.8 (−0.5; 3.9) *p* = 0.116
Height (m)	1.70 ± 0.07	1.76 ± 0.05	1.65 ± 0.04	0.11 (0.08; 0.15) *p* < 0.001
Weight (kg)	70.2 ± 11.8	75.6 ± 9.2	65.9 ± 12.1	9.7 (1.0; 18.4) *p* = 0.030
Body Mass Index (kg/m^2^)	24.2 ± 3.5	24.3 ± 3.2	24.1 ± 3.8	0.1 (−2.6; 3.0) *p* = 0.923
Foot size (European shoe size)	40.6 ± 2.3	42.6 ± 1.6	39.1 ± 1.3	0.1 (−2.7; 3.0) *p* < 0.001

Values are expressed as Mean ± Standard Deviation; between group differences are expressed as Mean ± Standard Deviation (95% Confidence Interval).

**Table 2:** Test–retest static posturography reliability estimates.

Variables	Mean ± SD	Absolute Error	ICC_2,1_ (95% CI)	SEM	MDC_95_
35–40 dB					
Oscillation Surface (mm^2^)	195.6 ± 117.2	53.4 ± 49.4	0.903 (0.787; 0.956)	34.9	96.7
Total Oscillation Length (mm)	373.6 ± 79.6	53.2 ± 39.8	0.837 (0.642; 0.926)	28.1	77.8
ML-Length absolute deviation (mm)	5.0 ± 2.4	2.9 ± 2.0	0.453 (−0.200; 0.751)	1.4	3.8
AP-Length absolute deviation (mm)	42.5 ± 13.8	4.4 ± 3.0	0.962 (0.917; 0.983)	2.1	5.8
LFS	0.65 ± 0.12	0.07 ± 0.07	0.840 (0.649; 0.927)	0.04	0.11
SVFAP	52.8 ± 35.7	7.9 ± 5.4	0.829 (0.625; 0.922)	3.8	10.5
ASV (mm/s)	50.4 ± 14.7	8.6 ± 5.8	0.871 (0.717; 0.941)	4.1	11.3
Mean Speed (mm/s)	12.4 ± 2.7	1.8 ± 1.3	0.839 (0.647; 0.927)	0.9	2.5
85–90 dB					
Oscillation Surface (mm^2^)	209.0 ± 124.5	77.6 ± 77.2	0.804 (0.570; 0.911)	54.5	151.0
Total Oscillation Length (mm)	378.8 ± 81.8	53.8 ± 40.0	0.859 (0.690; 0.936)	28.2	78.1
ML-Length absolute deviation (mm)	4.0 ± 3.0	2.9 ± 2.2	0.621 (0.168; 0.827)	1.5	4.1
AP-Length absolute deviation (mm)	44.5 ± 13.3	4.4 ± 3.7	0.952 (0.895; 0.978)	2.6	7.2
LFS	0.65 ± 0.12	0.07 ± 0.08	0.831 (0.628; 0.923)	0.05	0.13
SVFAP	37.4 ± 12.1	6.8 ± 5.5	0.873 (0.721; 0.942)	3.88	10.7
ASV (mm/s)	52.6 ± 14.9	7.5 ± 5.7	0.902 (0.784; 0.955)	4.0	11.1
Mean Speed (mm/s)	14.1 ± 7.6	1.8 ± 1.3	0.858 (0.689; 0.935)	0.9	2.5

SEM and MDC_95_ are expressed in the units described for each parameter. LFS: length as a function of the surface; SVFAP: speed variation as a function of Y; ASV: average of speed variation.

**Table 3:** Score differences between number of trials (2 and 3 mean average scores) for each acoustic condition (35–40 and 85–90 dB).

Variables	Differences between Mean Scores from 2 and 3 Measurements	
	40 dB	90 dB
Oscillation Surface (mm^2^)	0.5 (−60.5; 61.5) *p* = 0.986	4.7 (−59.0; 68.5) *p* = 0.881
Total Oscillation Length (mm)	4.9 (−36.7; 46.5) *p* = 0.815	4.6 (−38.9; 48.2) *p* = 0.833
ML-Length absolute deviation (mm)	0.1 (−1.1; 1.4) *p* = 0.805	0.2 (1.3; 1.7) *p* = 0.784
AP-Length absolute deviation (mm)	0.2 (−7.1; 7.6) *p* = 0.941	0.4 (−6.8; 7.7) *p* = 0.902
LFS	0.00 (−0.05; 0.07) *p* = 0.767	0.00 (−0.06; 0.07) *p* = 0.831
SVFAP	0.6 (−6.3; 7.4) *p* = 0.866	0.1 (−6.5; 6.8) *p* = 0.966
ASV (mm/s)	0.4 (−7.5; 8.3) *p* = 0.921	0.2 (−8.0; 8.5) *p* = 0.950
Mean Speed (mm/s)	0.2 (−0.7; 1.2) *p* = 0.817	0.2 (−1.3; 1.6) *p* = 0.833

Values are expressed as Mean (95% Confidence Interval) and *p* values.

**Table 4:** Static posturography score differences between acoustic conditions.

Variables	Differences between both Acoustic Conditions
Oscillation Surface (mm^2^)	8.1 (−50.3; 66.7) *p =* 0.780
Total Oscillation Length (mm)	5.5 (35.6; 46.6) *p =* 0.790
ML-Length absolute deviation (mm)	0.6 (−0.8; 1.9) *p =* 0.378
AP-Length absolute deviation (mm)	2.2 (−5.0; 9.5) *p =* 0.545
LFS	0.00 (−0.05; 0.06) *p =* 0.876
SVFAP	1.5 (−5.0; 8.1) *p =* 0.634
ASV (mm/s)	2.1 (−6.0; 10.1) *p =* 0.610
Mean Speed (mm/s)	0.2 (−1.2; 1.5) *p =* 0.797

Values are expressed as Mean (95% Confidence Interval) and *p* values.

## Data Availability

The data that support the findings of this study are available from the first author (S.O.C.-M) or the corresponding author (J.A.V.-C.), upon reasonable request.
